# Detection of hepatitis C virus among HIV patients in Port Harcourt, Rivers State

**DOI:** 10.4314/ahs.v21i3.8

**Published:** 2021-09

**Authors:** Glory Barinuaka Baeka, Julius Kola Oloke, Oluyinka Oladele Opaleye

**Affiliations:** 1 Department of Pure and Applied Biology (Microbiology/Virology Unit), Ladoke Akintola University of Technology, Ogbomoso, Oyo State, Nigeria; 2 Department of Medical Microbiology and Parasitology, College of Health Sciences, Ladoke Akintola University of Technology, Nigeria; 3 Department of Microbiology, Rivers State University, Port Harcourt, Rivers State

**Keywords:** Serology, HCV, HIV, CD4+ Count

## Abstract

**Background:**

Hepatitis C virus (HCV) a major human pathogen infecting millions of individuals worldwide, thereby increasing the risks for chronic liver diseases and has been discovered that HIV/HCV co-infected patients have a greater risk.

**Objective:**

To determine the prevalence of HCV infection among HIV infected people in Port Harcourt, Rivers State.

**Methodology:**

The patients were from the ages of 18 and above attending the antiretroviral clinic for over 6 months. The mean age of the participants was 36.91±8.38. Data were gotten from the 550 patients using a modified questionnaire and 5mls of blood samples were collected through venepuncture into EDTA bottles and spun at 3000rpm for 10 minutes separating the plasma from the whole blood. The CD4+ count was gotten from the patients' file and the samples kept at -700C till analized. HCV antibody was detected using a commercially available third generation kit manufactured by Melsin Medical Co and statistical analysis was done using a Stata version 16. P value was determined using ANOVA

**Result:**

Total number positive to the HCV antibody was 24(4.4%) of which 8(33.3%) were males, while 16(66.7%) were females. Prevalence (29.2%) was among patients in the 31–35 age range. The CD4+ count ranged from 22–864 cells/µl with a mean value of 303.08±194.

**Conclusion:**

From this study HIV/HCV co-infection occurs among HIV infected people in Port Harcourt. The CD4+ count was discovered to be low and was not age, nor gender dependent. HIV infected people should therefore be routinely screened for HCV.

## Introduction

Hepatitis C Virus (HCV) a major human pathogen infecting between 130 – 170 million individual worldwide, thereby increasing the risks for chronic liver diseases, which include steatosis, fibrosis, liver cirrhosis and hepatocellular carcinoma 1. HCV is transmitted via percutaneous contact with HCV infected blood, most commonly via shared injection drug use (IDU), contaminated blood products, or hospital equipment. The rates of mother-to-child HCV transmission generally are low but increase with maternal HIV co-infection 2. While the HIV infection is transmitted through blood and blood products, semen, breast milk and virginal secretion 3.

Acute hepatitis C (HCV) infection becomes chronic in 70% of patients, which represents a high rate of chronicity for a viral infection especially in patients with HIV infection 4. Concurrent infection of HCV occurs in about 20% of HIV infected persons worldwide^5^ of which the increased mortality in HIV/HCVco infected persons appears to be driven largely by accelerated liver disease 6. Hepatic disease has become the leading non-AIDS cause of morbidity and mortality among HIV- infected individuals after the availability of antiretroviral therapy (ART) became widespread in resource-sufficient areas of the world. In a 2006 multinational cohort of more than 25,000 HIV-infected persons in the United States and Europe, 14% of deaths were liver related and of those, 66% occurred in persons with concomitant HCV infection 7. Human Immunodeficiency Virus (HIV) infection and chronic viral hepatitis (Hepatitis B and C Virus infection) are global health problems of concern 8. In the African continent, the prevalence of the virus remains high in the sub Saharan region 9. In sub-Saharan Africa, HBV is the main cause of hepatocellular carcinoma (HCC), followed by HCV mostly as a result of chronic hepatitis with complications from liver cirrhosis 10, especially in HIV co-infected patients 11. Currently, about 10 million people are infected with hepatitis C in Africa^12^. In Nigeria, the current prevalence of hepatitis C infection is between 2.8% and 24.2% among adults from different sub regions of the county 13. In the South East Asian region alone, about 10 million people are infected with HCV 8. This suggest that the infection is much more in Asia than in Africa.

Divergent strains of the HCV genotypes1and 2, have been found to be endemic in West African countries including; Burkina Faso, Ghana, Guinea Bissau, Benin Republic and Nigeria. Genotype 3 is found in south Asia, genotype 4 in central Africa and Middle East while genotype 6 is distributed in south-east Asia 14. Genotype 5 has been found to be common in the northern region of South Africa and Belgium while genotype 7 has been reported only among the central African immigrants in Canada 15.

While the co infection prevalent rate in a study conducted in 27 countries in Africa as conducted by Rao et al 16 in which 4,2648 HIV positive patients participated, 101 were confirmed to be co infected with HCV/ HIV. The rate of fibrosis has been estimated to be 3 or more times higher in HIV/HCV co-infected patients, than in patients that are infected with HCV alone 17. HIV is also known to accelerate the progression of liver disease in HCV-infected persons because HCV replication increases in the presence of HIV resulting in elevated liver HCV RNA levels 18.

HCV infection has been suggested to negatively impact CD4+ cell count restoration and cirrhosis is associated with depressed CD4 cell counts, independent of HIV or HCV infection 19. Also the detrimental effect of HCV on HIV infection includes a significant reduction of CD4 cells and total CD4 percent 20.

Currently in Port Harcourt, there is little or no information on people co infected with HIV/HCV. This can be very dangerous since this can lead to death due to liver related issues in people co infected since they are already at risk health wise.

The objective of this study is to determine the prevalence of the occurrence of HCV among HIV infected people in Port Harcourt and also the effect of HCV on the CD4 count.

## Materials and methods study area

Rivers State which is one of the sates in the south southern part of Nigeria is multi tribal and a home to many oil companies. Port Harcourt, which is the capital of the state lies along the Bonny River in the Niger Delta region of Nigeria with its Coordinates: 4°53′23′N 6°54′18′E, covers an area of 360 km2 and consist of Obio/Akpor and Port Harcourt Local Government Areas. According to census 2016, Port Harcourt city local government area and Obio/Akpor local government area recorded a population of 1,382,592 and 878,890, respectively and land mass of 360 and 260 km2, respectively.

### Study population

The study population, were patients who attended the antiretroviral clinic at the Braith-Waite Memorial Hospital, Port Harcourt, and had been on antiretroviral drugs (HAART), who gave their consent. The study size was determined using the Cochran's formula.

### Study enrolment

The patients for this study were adults from the ages of 18 and above attending the antiretroviral clinic for over 6 months. Relevant data were gotten from the 550 patients between the months of April and October 2019 using a modified questionaire. The protocol used for the study was in adherence with the ethical standards laid down in the 1964 Declaration of Helsinki and its later amendments. All study subjects gave informed consent. Any subject who did not give consent was excluded.

### Ethical approval

Ethical approval for the study was gotten from the Rivers state ethical health committee

### Sample collection

Five mililiters of blood samples was collected through venepuncture into EDTA bottles to prevent coagulation. The samples were then spun at 3000rpm for 10 minutes to separate the plasma from the whole blood. The CD4 count was determined using a standard laboratory procedure with the use of a flow cytometer analyser (Version 2.4, Partec Germany) in which the freshly collected samples were placed on a haematological rocker and gently rocked for 4 minutes to homogenize the sample. Dispense 50 microliter of the blood sample into a rhoren tube and 20 microliter of CD4 monoclonal antibodies (piccoerythrin) and allowed to incubate for 15 minutes in a dark room after which a CD4 non lysing buffer is added and read using a flow cytometric analyser. The samples were kept in the ultra low at -700C till analysis was done.

### Serology

The detection for the HCV antibody was done using a commercially available third generation kit manufactured by Melsin Medical Co., limited. The assay was performed in adherence with the manufacturer's instructions. The optical density was read using the Emax endpoint ELISA microplate reader (Molecular Devices, California, USA) and the result was interpreted according to the manufacturer's instructions.

### Statistical analysis

Stata version 16 was used to analyze the data. The P value was determined using ANOVA.

## Result

Of the 550 HIV infected people tested of which 216 were males and 334 were females, 24(4.4%) of them tested positive to the antibody to the HCV virus, while 526(95.6%) tested negative.

Sex distribution of the HCV antibody among the study group showed that out of the 24 patients who tested positive to the HCV antibody, 8(33.3%) were males. While 16(66.7%) were females. There was no significant difference between the gender and the acquisition of the Hepatitis C virus, since the P value between the two was given to be 0.542. The CD4+ count ranged from 22–864 cells/µl with a mean value of 303.08±194.

People involved in the study were between 18 and 60 years. The prevalence of the virus antibody occurred among people in the age range of 31 – 35 with a percentage of 29.2% followed by those in the age range of 36–40(25%) (Table 1), with a mean age value of 36.91±8.38.

**Table 1:** Distribution of age group of participants

Age group	Numbers	%
18 – 20 yrs 21 – 25 yrs 26 – 30 yrs 31 – 35 yrs 36 – 40 yrs 41 – 45 yrs 46 - 50 yrs 51 – 55 yrs 56 – 60 yrs	1 - 4 7 6 3 2 - 1	4.17 - 16.67 29.17 25.00 12.50 8.33 - 4.17

Mean age: 36.91 ± 8.38

The CD4+ count for the patients ranged from 22–864 cells/µl with a mean value of 303.08 ± 194.29 (Table 2). There was no significant difference between the CD4+ count which has a mean value of 303.08 and age with a mean value of 36.91 with a P value of 0.526.

**Table 2:** Distribution of the mean CD4 count of participants

Age group	CD4 Mean	SD
18 – 20 yrs 21 – 25 yrs 26 – 30 yrs 31 – 35 yrs 36 – 40 yrs 41 – 45 yrs 46 - 50 yrs 51 – 55 yrs 56 – 60 yrs	186.0 - 213.75 326.57 369.5 153.0 409.0 - 453.0	0 - 59.32 282.13 162.52 35.17 239.00 - 0

Total CD4+ count mean: 303.08 ±194.29

## Discussion

Hepatitis C Virus which increase the risk of chronic liver diseases such as fibrosis, steatosis, liver cirrhosis, hepatocellular carcinoma etc especially in immunocompromised individuals 1 and mostly transmitted through blood transmission, aesthetic body piercing, drug injection and nosocomial transmission 21. In this study out of the 550 patients tested, 24(4.4%) were positive to the HCV antibody. This is below 23.3 reported by Mabayoje 22 in Oshogbo, 14.7% recorded by Balogun et al 23 in Lagos, 8.2% in Pakistan as discovered by Farooq et al 24. In the study carried out by Eze et al 25, an assessment of the prevalence of the Hepatitis C virus infection among HIV patients in a tertiary hospital in Nigeria was carried out of which the infected patients were confirmed using the ELISA technique. The subjects who were HIV positive were 240 of which 9(4.4%) of them were co infected with HCV/HIV and were recruited within one year. In 2012, Tremau Bravard 26 collected a medical data from 443 HIV positive patients in Abuja between September and May 2011 to detect the prevalence of the HCV antibody among them of which 35 patients were positive to the co infection of HCV/HIV. Also Hamza et al 27, did a cross sectional study among HIV patients at the Aminu Kano Univesity Teaching Hospital of which 440 adults participated and 7(1.6%) were co infected with HCV/HIV. These studies were all carried out to detect the prevalence of the infection of HCV among HIV patients. The result from this work agrees with 4.4% reported by Eze et al 25 in Benin. However, the result from this work is higher than 1.6% in North western Nigeria as recorded by Hamza et al 27 and 2.3% prevalence in Abuja as reported by Tremeau-Bravard et al 26. The reduction in prevalence with the finding in this work compared with most work done previously, could be due to awareness created from previous work done on the transmission of HIV for sterilization of sharp objects used for aesthetic purposes and other sharp objects and none reusing of used needles and screen of blood and blood products before transfusion, since the hepatitis C virus is mostly transmitted through these ways.

Although there was no significant difference between the both genders, but there was a higher number of females with the co infection than the male. This does not agree with the work done by Tremeau-Bravard et al 26 in Abuja in which the gender distribution was equal and in the findings of Hamza et al 27 in the north western part of Nigeria in which more males were infected than females but agrees with the report of Olmedo et al 28 in Rio de Janairo (Brazil) and Newton et al 29 in Ughelli. It is noted that females in the northern part of Nigeria are very conservative due to their religious beliefs which could be responsible for the low prevalence in the female gender than the male gender. From the findings of this study which agrees with those done in Ughelli and in Brazil, it is discovered that females are more liberated and involve in activities done by men such as involving in injectible drug, smoking with the usage of pipe which are means by which the virus can be transmitted coupled with other factors such as the usage of unsterilized aesthetic body piercing material which is common among the female gender than in the male gender and also through nosocomial transmission since women attend hospital more than men for various reasons. Hence the female tend to be more predisposed to the virus than the male. This is due to certain factors, which promotes the spread of the virus such as body piercing using unsterilized materials more common among the female gender than the male. This indicates lifestyle having effect on the spread of the virus.

The prevalent age range of people who tested positive to the HCV/HIV co infection in this study, was between ages 31 – 35 followed by ages 35 – 40 with a percentage of 29.2 and 25 respectively which agrees with the research carried out by Dhawan 4 but disagrees with the findings of Hamza et al. 27 in the North western part of Nigeria in which prevalence was among people of above 40 years old and in Pakistan according to the work done by Farooq et al. 24. The difference in the later studies could be due to geographical location.

The mean value of the CD4+ count was 303.08, which is an indication of a chronic co-infection of HCV/HIV since the patients have been on antiretroviral drugs for not less than 6 months, and should have begun to have an increase in their CD4 count, as observed by the McGovern et al. 19 in which he stated that HCV has a negative impact on the restoration of the CD4 count, which is associated with liver cirrhosis. Since the entrance of highly active antiretroviral treatment (HAART), non AIDS defining conditions have become major causes of illness and death in HIV infected patients. In particular, liver disease has emerged as a major cause of death in the HAART era 30. From the work done, it shows that the Hepatitis C virus co infecting with HIV, has a prevalence of 4.4% in the patients screened, showing that this percentage of people may be at a risk of developing liver related issues such as cirrhosis, fibrosis, hepatocellular carcinoma etc which may lead to speedy death among people co infected since they are already immunocompromised. Also, looking at the mean value of the CD4+ count of 303.08, shows that the co infection of HIV with HCV inhibits the recovery of the CD4+ cells since the patients have been on antiretroviral (HAART) for nothing less than six months. This puts people co infected at a risk of opportunistic infections, since the CD4+ cells play an important role in the immune system, especially the adaptive immune system. This study also discovered the prevalence of the co infection among the females than the male patients in this study shows that the females should be more hygiene conscious and sterilize sharp objects used for their aesthetic purposes in order help cub the transmission of the virus. The age range prevalence was between the ages 31 to 35, closely followed by ages 36 to 40 years. HIV patients should therefore be routinely screened for HCV and those discovered to be positive should be placed early on antiretroviral medication to control the replication of the virus thereby preventing death from liver related issues in HIV/HCV co infected patients.
